# Determinants of mistrust in digital health research and approaches to address them among Muslim ethnic minorities living in the United Kingdom: a qualitative study

**DOI:** 10.1186/s12939-025-02583-3

**Published:** 2025-08-15

**Authors:** Syed Mustafa Ali, Mohammad Mahin Saiyed, Aneela McAvoy, Robert Mackin, Caroline Jay, Sabine N. van der Veer

**Affiliations:** 1 Division of Informatics, Imaging and Data Science, Centre for Health Informatics, Manchester Academic Health Science Centre, The University of Manchester, Manchester, UK; 2 National Institute for Health and Care Research (NIHR) Applied Research Collaboration – Greater, Manchester, UK; 3 Anjuman-E-Islamia, Newham, London, UK; 4 School of Social Sciences, University of Manchester, Manchester, UK; 5 Department of Computer Science, School of Engineering, University of Manchester, Manchester, UK

**Keywords:** Digital health, Health inequity, Trust in health research, Inclusive research, Widening participation

## Abstract

**Background:**

Under-representation of Muslim ethnic minorities and their mistrust in health research are known barriers to achieving digital health equity. Therefore, this study aimed to understand determinants of Muslim communities’ mistrust in digital health research and explore potential approaches to address this and increase their participation in health research.

**Methods:**

This study employed a constructivist grounded theory design, involving focus groups with Muslim ethnic minorities living in the United Kingdom. We conducted nine focus groups in mosques, co-moderated by a digital health researcher and an Imam.

**Findings:**

Muslim ethnic minorities had several negative perceptions about digital health research, which were mainly influenced by lack of their awareness about the purpose and conduct of research. They felt excluded from health research and did not perceive taking part as beneficial to them or their community. These were exacerbated by how research findings related to Muslim ethnic minorities in the UK were used or shared in public spaces (e.g., by media outlets or healthcare providers). Participants suggested that Imams and mosques could play a role in addressing these negative perceptions by raising awareness among their communities using digital resources (e.g., bite size videos, social media community groups) and during regular gatherings.

**Conclusion:**

Negative perceptions about health research are common among Muslim communities, which are further exacerbated by the way research findings related to South Asians are discussed in public spaces. Despite this, there is a potential of building the Muslim community’s trust and improve their participation in health research if health researchers work collaboratively with mosques or Imams and leverage community-based networks and resources.

**Supplementary Information:**

The online version contains supplementary material available at 10.1186/s12939-025-02583-3.

## Introduction

During the COVID-19 pandemic, there was an increased recognition of ethnic health inequities worldwide, as well as in the United Kingdom. During the same time, the introduction of digital health technologies into care pathways was widely promoted to access healthcare services, which created new or exacerbated existing health inequities [1], referred to as ‘digital health inequities’. The NHS Race and Health Observatory promotes the use of digital apps or services to reduce existing health inequalities, however, it also recognises how shortcomings in accessibility of digital tools may result in unintended consequences for certain groups [2]. To avoid these unintended consequences, we need to develop digital health technologies that are equitable and have potential to address existing health inequities [3, 4]. However, developing digital health technologies is not possible without improving participation and engagement from the diverse sub-groups of the population [5–7].

The underrepresentation of ethnic minority groups in (digital) health research is significant, and the same groups normally face inequities in health [8–10]. Owing to the underrepresentation, the lack of health data from these ethnic minority groups is recognised as one of the most significant barriers in promoting or developing evidence-driven digital health technologies [11]. For example, underrepresentation of Black and Asian ethnic minorities in medical and health research [12] may likely to perpetuate or even widen the existing gaps in digital literacy and access to health services [12].

Underrepresentation of ethnic minorities in health research stems from the issue of mistrust in it. Trust plays a critical role in shaping individuals’ willingness to engage with health systems and participate in health research. As Gilson [13] highlights, trust is central to both effective care and relationships with health professionals and institutions. Drawing on the social theories of Giddens and Luhmann [14], trust can be seen as a way of managing uncertainty, where individuals place confidence in systems they do not fully control or understand. In health research, trust is seen both as relational and institutional built on shared values and cultural practices, which are often shaped by religion among Muslims globally, including in the United Kingdom. Muslims are an ethnically diverse group, including Asians, Africans, Arabs, Turks, etc. [15]. Mistrust in health research, being a cause of underrepresentation of these groups, is attributed to accessibility and language barriers [16]. Therefore, promoting trust in health research to increase the diversity of research participants is one of the recommendations given by the National Health Services, England [17]. Similarly, building trust is also an important consideration for developing digital health technologies [2], for which the role of trusted advocates from target communities is equally important [17].

Many ethnic minority groups in the UK are Muslims, and mosques hold a central place for them. Imams are religious scholars, and majority of Muslims trust their words and actions [18, 19]. Leveraging the existing trust relationship between Imams and worshippers, mosques have been engaged before for health promotion and recruitment for research studies [20–23]. However, it is unknown regarding what trust in health research entails, and how it is manifested in the digital health context.

Therefore, this study aimed to understand determinants of Muslim communities’ mistrust in health research and digital technologies and explore potential approaches to build trust among Muslims to promote their participation in health research. The findings of this study will help researchers and technology developers learn about the potential approaches for working with Imams and mosques to build Muslims’ trust and improve their participation and engagement in digital health research.

## Methods

This study employed a constructivist grounded theory design [24] to emphasise that realities are socially constructed. In our study, we explored ‘trust and participation in research’ as a reality and explored its linkages with a social context, i.e., connectedness among Muslim community members and the role of mosques as a place where people are connected socially as well as perform religious activities, which are led by Imams. We reported our study in accordance with Consolidated Criteria for Reporting Qualitative Research [25] (see annex A for a completed COREQ checklist).

### Public involvement group

We organised three virtual public involvement group meetings, each with up to eight members of the public associated with faith-based organisations. AM moderated the group discussions. The group’s suggestions informed the design and conduct of our research, including recruitment strategies, data collection methods, and routes and formats for disseminating findings.

### Participants and recruitment

Using convenience sampling, we invited adult community members, including local Imams, administrative committee members, and Muslim worshipers to participate in face-to-face discussions in their local mosque. Mosques were conveniently selected based on researchers’ (SMA, MMS) personal and professional networks. All mosques were located in areas with a high proportion of South Asians. Adults who were able to speak English, Urdu or Punjabi and willing to attend in-person focus groups were considered eligible. As mosques are predominantly attended by male members, we encouraged interested Imams to help organise mixed-gender group discussions in a religiously and culturally appropriate manner. Such biases in recruitment via mosques are highlighted previously [26]. The study was promoted through announcements during prayer congregations and in mosque-managed WhatsApp groups. Interested and eligible people read the participant information sheet and could ask questions before providing written informed consent.

### Data collection and analysis

Consented participants completed a brief baseline questionnaire prior to joining a focus group discussion. Two researchers (SMA, MMS) moderated all focus group discussions. SMA is a digital health researcher with experience conducting focus groups for digital health and public health research. MMS is an active Imam, with an interest in promoting Muslims’ participation in research. Both researchers were male and of South Asian ethnic backgrounds, and were proficient in understanding and speaking English, Urdu and Punjabi.

Informed by literature [27–31] and inputs from the public involvement group, we developed a focus group guide (Annex B), covering topics such as people’s awareness of and opinions about research, religious influences, factors that affected their trust in research, and approaches to further engendering trust. In addition, visual prompts were used to support communication and enhance data quality and validity [32]. For example, we used a graphical representation of a typical process of taking part in research, starting from announcing a study to collecting data for a study (Annex C) and showed examples of a study flyer, a participant information sheet and a consent form as common participant-facing research documents. Both researchers took notes during the focus groups, which helped guide the group discussions and post-discussion reflections. In line with constructivist grounded theory methodology [33], the researchers held several debriefing and reflection meetings to improve data collection, analysis and interpretation. Insights from these meetings informed refinement of the topic guide, clarification on emerging ideas captured in earlier focus groups, and co-creation of strategies to build trust in health research. We also used earlier identified strategies or recommendations as prompts in later focus groups to iteratively validate and refine them. Lastly, the codebook was developed and refined iteratively by reflecting how researchers’ unique positionalities influenced the coding and interpretive processes.

Focus groups lasted between 40 and 60 min, and data collection continued until data saturation was achieved, and no further clarification is needed on generated ideas or themes. All focus groups were organised in local mosques at a time convenient for all participants, audio-recorded, and transcribed verbatim by a professional service. Transcription and translation in English of parts of the audio-recordings were done by a researcher (SMA) who understood Urdu and Punjabi, which he later anonymised prior to coding and analysis in NVivo (v12).

SMA and MMS reviewed and coded the transcripts line-by-line and developed a coding structure, which was refined iteratively and inductively. After each focus group discussion and reviewing a transcription, SMA and MMS had a debriefing meeting to identify and record emerging ideas and adapt the topic guide and data collection approach accordingly. They used their own positionality to interpret the focus group data. We presented the determinants shaping mistrust in digital health research as themes and illustrated them with participants quotes. We also identified key recommendations for mosques and research institutes for building trust and improving participation in digital health research among Muslims.

## Findings

### Participant characteristics

Sixty-six adult Muslims participated in nine focus group discussions organised in their local mosques across six cities in the UK. The majority of participants were male (*n* = 58; 88%), younger than 35 years (*n* = 42; 64%) and of Pakistani ethnic background (*n* = 57; 86%). Participants’ characteristics are presented in Table 1. The public involvement group also recommended disseminating findings regarding perceived causes of mistrust in health research and potential approaches to address them (see annex D).

**Table 1:** Characteristics of participants (*n* = 65)

Variables	Number (percentage)
***Gender***	
Male	58 (88)
Female	8 (12)
***Age (years)***	
18–24	23 (35)
25–34	19 (29)
35–44	14 (21)
45–54	7 (11)
55 and above	3 (4)
***Ethnicity***	
British Pakistani or Pakistani	57 (86)
British Indian or Indian	6 (10)
Other^a^	3 (4)
***Education***	
College or University	33 (50)
Post-graduate	12 (18)
Further education	10 (15)
Secondary school	10 (15)
Prefer not to say	1 (2)
***Employment status***	
Full time employed	31 (47)
Student	13 (20)
Self-employed	9 (14)
Part-time employed	8 (12)
Unemployed or volunteering	5 (7)
***Type of participants***	
Community member	38 (58)
Administrative committee member^b^	18 (27)
Imam	10 (15)

^a﻿^Included British Kashmiri, Arab and white British

^b^These members are volunteers from the community, who look after day-to-day affairs of mosques

### Determinants shaping mistrust in digital health research

Based on the thematic analysis of the focus group discussions, we identified three themes related to determinants which may shape Muslim’s (mis)trust in digital health research. These included, (a) conception of health research in the context of other systemic inequities; (b) perceptions of being excluded; (c) perceptions that taking part will not benefit them or their community.

####  Conception of health research in the context of other systemic inequities

Overall, participants had limited experience of taking part in health research. For nearly all of them, this was their first ever health research study. Many mentioned that, historically, their mosque had been involved in raising awareness about health conditions and providing health services (e.g., vaccination, screening), but inviting people to participate in health research was found to be rare. One participant said:

“*This is the first research group in this mosque, but in the past we had so many different medical teams who used to come here regularly to talk to people, like so many”.*

Historic events related to unethical health research might have prevented Muslim ethnic minorities to promote and participate in health research as one of the participants said:

*“…I’m not a conspiracy theorist, but sometimes you don’t have to be a conspiracy theorist for a conspiracy to be proven true. For example, some research was conducted without the knowledge of somebody [referring to news about research in Africa]”*

On the other hand, one of the participants mentioned a potential barrier as:

*“…the South Asian community here…when…any new thing is happening…most of the people think the agenda’s setting against us…they have such mindset regarding this”*

Another participant shared his opinion, which can be attributed to general concerns about health research data:

*“How does the general public in the UK trust the data being collected. So, it's not just something specific to the [South] Asian community, some of the grievances, the apprehensions people have…how your data is being collected, who's it being shared by, you know, all these fears…so what's happening with that data. So, those grievances are there, and I think they're probably more important than the cultural ones”*

Without having first-hand experiences of research participation, the media and public opinion seemed to have played important role in forming people’s—often negative – perceptions about research, digital technology (such as artificial intelligence) and research data/findings. Some participants explained this as follows:

*“The media outlets tend to publish these papers and they misinform so that’s one of the key things, for example, the situation with the vaccines…there is a mistrust”*

*“You hear something…on the mainstream media…as an example, all about AI… everyone watches the mainstream media, and are aware of it, et cetera. So, they might have some apprehensions from what they see in the mainstream about technology, and then they’ve taken that on board, and maybe when approached regarding, sort of, research, they might think, well I've seen something on the news and it says, don't do this”.*

Participants also highlighted how being invited to participate could be embedded in people’s past experiences.

*“I don’t think that’s…with health, I think a lot of times they’re stating facts but because the Asians, a lot of us, not all of us, have such a big ego that everything…and because of what’s happened in the past as well with the experience here of racism, everything is racist to them…”*

In addition, participants perceived that there were certain stereotypical behaviour or attitudes in healthcare settings that might have resulted in negative perceptions about heath research and how findings were used to inform clinical practice. For example, one participant gave this example:

*“…I always get asked this from my parents, about diabetes research. Now, if you go to a local doctor, he'll tell you, 60, Asian…diabetes guaranteed. But when you look at your grandparents, they didn’t have diabetes, so what do you mean, it's guaranteed? So, we find sometimes the research is done in such a way that, it negatively impacts our communities”*

#### Perception of being excluded

Linked to a lack of opportunities to participate in health research, there was a strong perception of being excluded. Several participants thought that this was because of their occupation and educational background:

*“I think the lack of opportunity is one… if you’re working in a certain occupation, you might not have that access compared to somebody who’s probably in a university setting or in a research setting. For example, working with NHS [National Health Service], it might be different to somebody who’s maybe like an Uber driver…When it comes to research purposes, they might not get the same opportunity”.*

*“if…you have an educational background, in the sense that you’re part of a university or you’ve graduated from university, these people are probably more inclined to participate…as opposed to perhaps somebody who… let’s say when they were 18, they went into work straightaway”.*

In addition to a lack of opportunities, language was perceived as a major barrier, particularly for older people and women because many may not use digital technology and/or may not be able to read, write and communicate in English:

“*…a big problem we have is, elderly people, generally, are not able to access it [technology]. And it actually puts them off…and I think that gets missed in the data that’s represented…and only what compounds the problem is, our elderly sometimes can't read English as well…”*

*“…many women in our communities, generally still tend to have traditional roles, so they're staying at home…so, perhaps, looking into…Zoom meetings, and the like, would be better to conduct research with them. As opposed to hoping that they'd be able to turn up to a mosque, or a centre, or a church, and then speak to them there”.*

In contrast, some said that living within a household or a family of multiple generations reduced their reliance on their first language and increased their use of English, potentially addressing the language barriers to research participation mentioned by other participants:

*“It is a hard work communicating with my own children in Urdu. The only time I use Urdu is when I speak with my beloved mother and my elderly relatives out of respect and out of the etiquette. Other than that, my brothers and sisters…my kids, my nephew and nieces all are English (speaking), so if that’s happening in my household, I think that’s absolutely evident throughout the whole country…”*

#### Perception that taking part will not benefit them or their community

Participants shared general perceptions about how their community views health research and invitations to take part in it:

*“…for example, [if] it’s someone who is non-Muslim, he wants to come to the mosque and he presents himself in front of the people, they’re [people are] going to be, like, oh, they’re [researchers are] just doing it for their own benefit…they’re not going to have that trust.”*

*I'm not talking to you [researchers conducting focus group] but, you know, when someone comes to you and says, I’ve got research, what’s the…you know, somebody wants to prove a point, is that right, and they're trying to build a caseload, they're trying to build evidence in order for their objective to be fulfilled.*

Participants had general perception that participating in research might not be valued and nothing would change as a result of their participation. One participant mentioned it as:

*“You think that you're going to get under d or not valued enough and…so therefore you'd rather not speak”*

Participants also perceived lack of transparency in research procedures and thought that their participation and data might be exploited for researchers’ own benefits. A participant mentioned how people might think about participating in health research:

*“thinking of…data leakages, I think…is it a societal thing at the moment that…we don’t know what’s going to happen to our…not just data but…yeah, our views and whether those views can be used to…like, what’s your [research’s] ulterior motive?”*

### Approaches to building trust and promoting participation in (digital) health research

Based on the focus group discussions, we identified socially-constructed recommendations for mosques/Imams and research institutes to consider approaches to build trust and promote participation in (digital) health research among Muslim minorities or in particular South Asians living in the UK.

#### For mosques and Imams

Participants identified the role of imams as important in building trust and promoting participation in health research, since mosques are recognised as organisations mandated by the community to work for the community’s benefit. Key recommendations along with illustrative quotes are given below in Table 2:

**Table 2:** Recommendation for mosques and Imams

Recommendation	Illustrative quote
Trust in Imams should be leveraged to build the community’s trust and promote their participation in health research.	‘…I think the way you’re going to now conduct this particular research in different masjids [mosques], the only reason why we’re here is because the Imam said so. And I think that’s the most compatible way of doing this…to approach the Imam to approach the people…Speak to the Imam to get people to come and they’ll come because people trust the Imam.’
Mosques should set up working groups under the leadership of Imams to support different research related activities.	‘I was thinking that you could start from a very small group where you have that message across, and then that group can own the participation and then they can spread that message into their own contacts. And that’s how I think you can spread it’
A network of mosques should be established to increase research awareness and participation at a wider scale.	‘.…it would be more effective if there’s a contribution, not just from one mosque, but multiple mosques that talk about the same topic. It would create more awareness…if everyone starts talking about it, they’re going to start listening, they’re going to start going to these researches, and promoting it for the benefit of the Muslim community
Five-time prayers and Friday prayer gatherings should be used to promote participation in health research.	‘Education is a main thing…people need to change their mindset. A lot of the elders you will not get through to them, irrespective, whatever you do…WhatsApp or things on the note board, people are going to come in and not take any notice. Jumma [Friday prayer], they’ll hear it…that is the only communication that will get through to them
Current social media groups managed by mosques or social media in general should be used to disseminate information related to research, including invitation to participate.	‘…social media plays a massive in our day to day lives. Everyone’s got a mobile phone…And obviously you know when you’re trying to reach out to youngsters in particular…everyone’s on social media’
Educational classes and social gatherings for youth and women should be used to raise awareness about research and provide opportunities to participate in research.	‘I think we underuse the ladies’ category…the ladies are very willing to do these things voluntarily, because they are not – I don’t mean it in a bad way – they are not there just for the kitchen or washing your clothes and all that, they will actively come more to do this than anybody else. I can give an example, that we have exercise classes on Saturdays and we have ladies coming in from [area name], purely because there is no other place who does the exercise classes for ladies’

#### For researchers and research institutes

Credibility of health researchers and research institutes they belong to are important considerations for building community’s trust in health research. Specific recommendations and associated quotes are given below in Table 3 along with the determinants of mistrust they may likely to address:

**Table 3:** Recommendation for researchers

Determinant of mistrust	Recommendation	Illustrative quote
Conception of health research in the context of other systemic inequities	• Researchers should consider raising awareness in communities regarding health research, their types and benefits • Researchers should consider explaining health research and data protection principles to potential participants (e.g., in a presentation, or video) before sharing other participant-facing materials with them • Researchers should prepare bite-size information in an audio-visual format to raise awareness about health research and encourage people to take part • Researchers should work with Imams and consider disseminating the results of research in which participation was sought previously from Muslim communities	‘there needs to be a greater level of transparency, and bringing the discourse down to a simple man's level, so that everyone understands what the research is about’ ‘…is it going to be face to face, where you might have some slides that explain it, and going through it, and have a Q&A before you start any, sort of, chat. I think that way of engaging individuals. Some people will read through it and say, fine, I understand….there’s a lot of anecdotal fear…If it’s explained openly as you are doing at the moment to say that the information will be anonymised, the information is for medical research…’ ‘…[referring to a participant information sheet used as a prompt] if you’re going to write paragraphs and paragraphs on…then I don’t think it would work. But what would work is perhaps like if it’s bitesize information. For example, videos definitely work, like short clips’ ‘…you [researcher] are going to get the data and you [researcher] are analysing it, you [researcher] are going to come to the conclusion. So, you [researcher] can pass the message onto him [Imam], but I think we want that from you [researcher], that coming back. That’s what your participation is.’
Perception of being excluded	• Researchers should consider improving the Muslim community’s digital skills to enable participation in digital health research involving the use of digital devices, such as smartphones and smartwatches • Researchers should collaborate with Imams and mosque working groups to plan research participation from the community, e.g., providing translation support to people with language barrier, or explaining research to potential participants • Research teams should consider bilingual team members from Muslim backgrounds to address language barriers as well as building their trust and confidence • Researchers should consider compensating research participants for their participation, e.g., in the form of gift voucher	‘You’ll need to upskill the community…because…they might not understand what you actually have to do or how they can use it [technology]. And if they don’t, they’re not going to use it [technology]’ ‘You placed a paper consent form here and we read it…so if those elderly people were sitting here, they would get up straightaway and leave, or they would not have a clue. Not because they don’t understand English, because some people need a briefing as well. That helps to build a bit of trust as well’ ‘If universities need to get the research from the older people or the data…they need to get somebody who’s speaking their language, because they don’t want to come out from their comfort zone. So that way, they will trust more because somebody is speaking their language, they’re bound to maybe answer’ So I think…it’s the human condition, isn't it…We…especially in the society we’re living in, Amazon’s [gift voucher] got a lot to do with it…
Perception that taking part will not benefit them or their community	• As a part of the invitation to take part, researchers should verbally communicate the benefits of research participation for potential participants and their community • Researchers should present some examples to demonstrate how participation in research has translated into improved health outcomes for the community. This would help building confidence among community members in health research	‘There has to be something at the end for you to feel fulfilled …that I took a part in…there was some benefit for me or for the community, there was some fulfilment at the end’ ‘But if you say for instance as an example, look, we know that…from research that’s already been done in the black community that the black community in general are predisposed to sickle cell disorder and as a result of that, this is what we did, these are the extra services we made available…that is how you get people to take part in it.’

## Discussion

Several factors were perceived to be shaping Muslim communities’ mistrust in digital health research, particularly by South Asian Muslims, but lack of opportunities to participate in health research seemed to have had the greatest influence, which led to a lack of awareness about research procedures. Negative perceptions were exacerbated by how research findings related to Muslim ethnic minorities in the UK were used or shared in public spaces, (e.g., media outlets and healthcare organisations). This may have led Muslim ethnic minorities (including South Asians) to have concerns and negative perceptions about health research. This study further highlighted the potential role of Imams and mosques in addressing these negative perceptions by raising awareness among communities with the use of digital resources (e.g., bite size videos, social media community groups) and during regular gatherings. There is a potential to build the Muslim community’s trust and improve their participation in digital health research if health researchers and their organisations work collaboratively with mosques or Imams.

Trust is one of the important factors crucial for ensuring improved participation from ethnic minorities [17, 30, 34], which our findings confirmed. Also, a lack of trust may intersect with language barriers and accessibility challenges faced by ethnic minorities [17], therefore, addressing trust along with other barriers is important for inclusive research. We found perceptions of being excluded or discriminated against to be important barriers to participation in digital health research, which are also important for addressing barriers related to accessing health services and reducing health disparities [27, 28, 30]. Since trust or mistrust in health research can be manifested differently in different social and cultural context [17, 31, 34], such contexts of target communities should be considered while addressing mistrust [28]. For example, understanding of research principles (e.g., data protection) and research procedures (e.g., recruitment strategies, dissemination formats and routes) should be co-developed with ethnic minorities to build the community’s trust in health research [35].

In our study we found that lack of opportunities to take part in health research led to lack of awareness about research procedures and research-related terminologies. During the group discussions, we had to describe key concepts (e.g., data protection, informed consent) or the purpose of research activities (e.g., digital health research and data sharing) to prompt participants to share their views on how and what changes in the conduct of research could potentially improve their understanding and trust in health research. Therefore, by raising awareness, the negative perceptions about health research can potentially be addressed. The same approach has been developed and tested, which focused on increasing awareness by local champions with the help of seminars, audio-visual content and opportunities to ask questions directly from researchers [36]. Similarly, mosques have also played a role in raising community’s awareness about important health issues [37]. As we found in our study, there is a need for raising general awareness about health research and benefits of participating to improve representation of Muslim ethnic minorities in health research. However, future research should identify the information needs of diverse Muslim population subgroups based on their socio-demographics and explore ways to address them.

A strength of our study was having an Imam as the focus group moderator, which encouraged participants to share their views openly. However, this may have deterred individuals who do not attend mosques or trust Imams, potentially leading to an overestimation of their role in promoting health research. It is important to note that the mosques where we recruited participants were predominantly attended by male Muslims from South Asian heritage. This may limit the generalisability of our findings to other Muslim minorities living in the UK, such as Black Africans or Caribbeans, as well as to women. Although our participants suggested challenges faced by some of the other sub-groups (such as women or older adults), future digital health research in Muslim ethnic minorities should employ a variety of recruitment routes to increase the diversity of study samples and perspectives. For example, by combining recruitment via mosques with recruitment via charitable organisations supporting ethnic minority women in the community.

## Conclusion

Perceptions of being excluded and research lacking benefits for the community are determinants of mistrust among Muslim ethnic minorities living in the United Kingdom. These negative perceptions are further exacerbated by lack of opportunities to participate in research and the way research findings related to South Asians are discussed in public spaces. However, Imams may have a potential role in addressing the issue of mistrust in digital health research among Muslims. Imams and mosques may support raising awareness about digital health research to address prevailing misconceptions and to build the community’s trust in digital health research. This will ultimately result in wider participation in health research from Muslim ethnic minorities. Community-based networks, such as regular gatherings, provide an opportunity to promote research, disseminate results, and address existing barriers to inclusive participation, such as addressing language barriers, and digital literacy. Researchers, technology developers and research institutions should consider developing partnerships with Imams and mosques in addressing barriers to inclusive digital health research and ultimately addressing ethnic health inequities through improved participation.

## Supplementary Information

Supplementary Material 1.

Supplementary Material 2.

Supplementary Material 3.

Supplementary Material 4.

## Data Availability

Anonymised data will be made available by the corresponding author upon a reasonable request.
