# Supplementary material for: Determinants of mistrust in digital health research and approaches to address them among Muslim ethnic minorities living in the United Kingdom: a qualitative study
Source: Int J Equity Health. 2025 Aug 15;24:225. doi: 10.1186/s12939-025-02583-3 (PMC12357475; doi:10.1186/s12939-025-02583-3)
Supplement: Supplementary file 2 — Supplementary Material 2. [file 12939_2025_2583_MOESM2_ESM.docx]

**Focus Group Guide**

Note: A public involvement researcher (i.e., an active Imam) will moderate discussions alongside a digital health researcher (from the University of Manchester) who will also take notes

What is your opinion about health research and participating in health research in the UK? (*concerns opinion about research*)

- Explore previous experience of taking part in research
- *[Researchers will use a visual prompt to describe a process of participating in a research by using an actual digital health study and showing all study resources e.g., consent form ,PIS]*
- Explore whether and how/why involvement of technology in research changes their opinion about research participation
- Whether and how participation in research helps improving health outcomes?

In your opinion, what is the influence of religion in health research participation? *(concerns religious influence)*

- Explore how the positive influence can be further improved
- Explore what role Imams can play in creating that influence
- Explore the role of mosque
- What information or activities are needed to create that influence?
- Any specific considerations related to health technology development and research and its place in/relation with religion

In your opinion, why Muslim ethnic minorities do or do not take part in health research? *(concerns determinants of trust or distrust)*

- Explore determinants, such as trust issues, language barrier, access issues [facilitator will note down determinants in a template]
- Explore if determinants change or remain the same if research focuses on digital health technology as a research topic/area
- What information do you or your peer have about research?
- How information is disseminated among Muslim peers? Any thing about research? Explore cultural role in dissemination of information
- How information is tested if it is valid?

How participation of Muslim ethnic minorities can be promoted in research? *(concerns approaches to build trust)*

- *[Researchers will use a visual prompt to describe different phases involved in a typical digital health research and moderate discussion in accordance with that]*
- Explore different actions or steps that researchers or tech developers may take for each phase of a research
- Explore the role of mosques and Imams in promotion of research
- How trust in health technology and research can be built? Think about specific resources
- Think and discuss potential approaches
