# Supplementary figures and images for: Determinants of mistrust in digital health research and approaches to address them among Muslim ethnic minorities living in the United Kingdom: a qualitative study
Source: Int J Equity Health. 2025 Aug 15;24:225. doi: 10.1186/s12939-025-02583-3 (PMC12357475; doi:10.1186/s12939-025-02583-3)

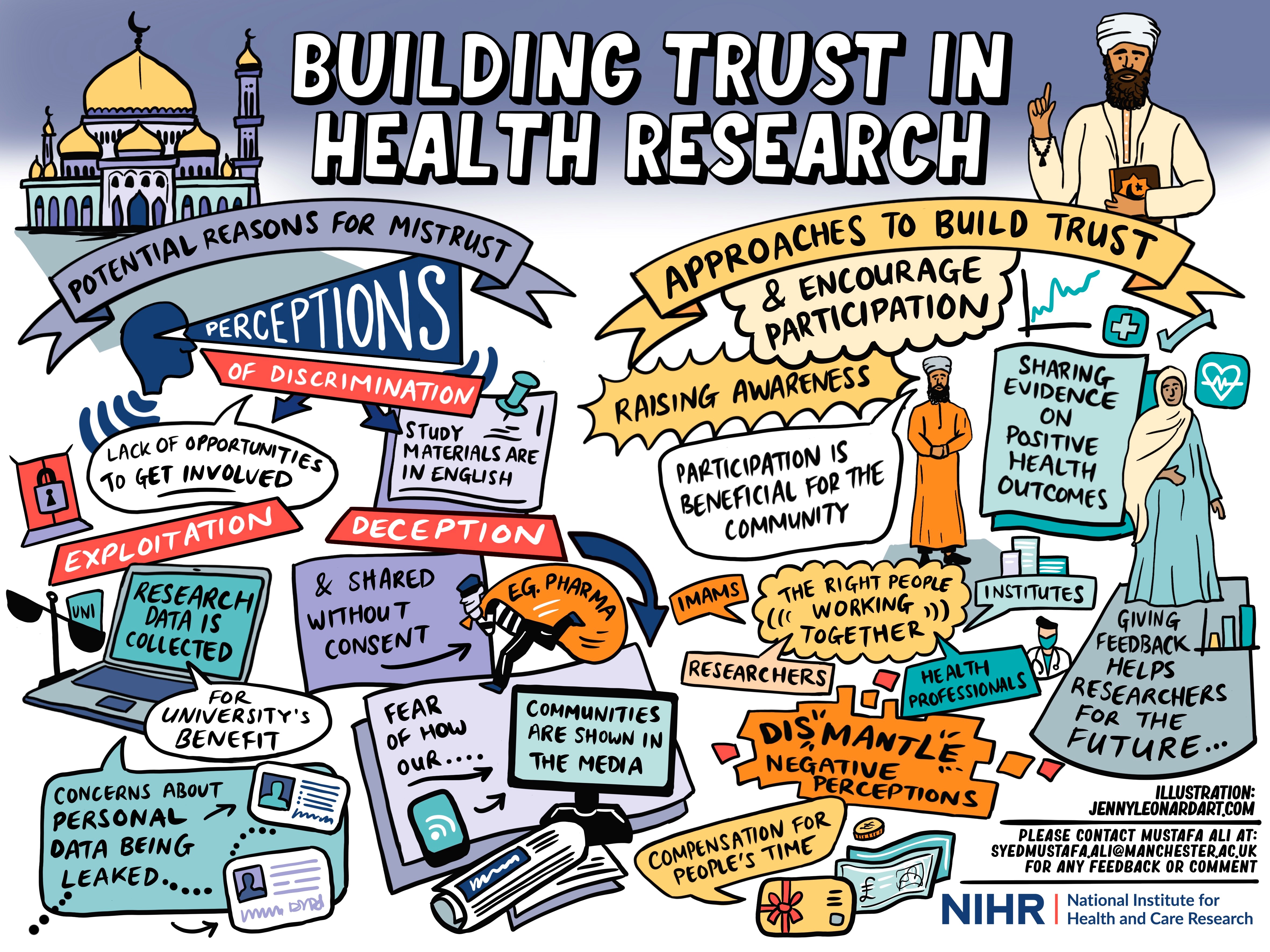

Supplement: Supplementary file 4 — Supplementary Material 4. [file 12939_2025_2583_MOESM4_ESM.jpg]
