# Supplementary material for: Determinants of mistrust in digital health research and approaches to address them among Muslim ethnic minorities living in the United Kingdom: a qualitative study
Source: Int J Equity Health. 2025 Aug 15;24:225. doi: 10.1186/s12939-025-02583-3 (PMC12357475; doi:10.1186/s12939-025-02583-3)
Supplement: Supplementary file 4 [file 12939_2025_2583_MOESM3_ESM.docx]

The University of Manchester

ABouT Research: Approaches to Build Trust in Research
Visual prompt: Process of taking part in research

Announcing the study

Study flyer	To let people know briefly about the study, and how to get in touch

↓

Expressing interest

E-mail or telephone call or in-person	Express your interest to take part in a study

↓

Screening

Screening proforma	Screen a person for his/her eligibility

↓

Knowing more about a study

Participant information sheet	What is the study, why we are doing it and what we are expecting from you

↓

Consenting

Consent form	Basis on which you agree to take part

↓

Data collection begins
